# Prevalence and psychopathological characteristics of depression in consecutive otorhinolaryngologic inpatients

**DOI:** 10.1186/1472-6815-11-7

**Published:** 2011-08-31

**Authors:** Thomas Forkmann, Christine Norra, Markus Wirtz, Thomas Vehren, Eftychia Volz-Sidiropoulou, Martin Westhofen, Siegfried Gauggel, Maren Boecker

**Affiliations:** 1 Institute of Medical Psychology and Medical Sociology, University Hospital of RWTH Aachen, Pauwelsstraße 30, 52074 Aachen, Germany; 2 Dept. of Psychiatry and Psychotherapy, LWL-University-Clinic, Ruhr-University Bochum, Alexandrinenstr. 1-3, 44791 Bochum, Germany; 3 Institute of Psychology, University of Education Freiburg, Kartäuserstr. 61b, 79117 Freiburg, Germany; 4 Clinic for Otorhinolaryngology, University Hospital of RWTH Aachen, Pauwelsstraße 30, 52074 Aachen, Germany

**Keywords:** prevalence, pathopsychology, depression, otolaryngologic inpatients, otorhinolaryngology

## Abstract

**Background:**

High prevalence of depression has been reported in otorhinolaryngologic patients (ORL). However, studies using a semi-structured interview to determine the prevalence of depression in ORL are lacking. Therefore the present study sought to determine the depression prevalence in ORL applying a semi-structured diagnostic interview and to further characterize the pathopsychological and demographic characteristics of depression in these patients.

**Methods:**

One-hundred inpatients of the otorhinolaryngologic department of a German university hospital participated voluntarily (age M = 38.8 years, SD = 13.9; 38.0% female). Depression was assessed using a clinical interview in which the International Diagnostic Checklist for depression (IDCL) was applied. Patients completed the Brief Symptom Inventory (BSI) which constitutes three composite scores and nine symptom scales and the Beck Depression Inventory (BDI). Multivariate analyses of variance, correlations and effect sizes were conducted.

**Results:**

A prevalence of depression of 21.0% was determined, 38.0% of the depressed patients were female. Depressed patients showed higher scores on the BSI-scales "interpersonal sensitivity", "depression", "anxiety", "phobic anxiety" and "psychoticism" with medium effect sizes.

**Conclusions:**

High prevalence of depression was found which is in accordance with results of prior studies. Depressed patients showed higher psychological distress as compared to non-depressed patients. The results call for carrying on in engaging in depression research and routine depression screening in ORL.

## Background

Depressive disorders are among the most prevalent mental disorders of our times [1,2], coinciding with increased symptom burden, functional impairment, and immense socio-economical costs. Prevalence was found to be especially high in populations of patients with physical diseases [3-6]. Furthermore, depression may affect the course of comorbid physical illnesses and worsen their outcomes [5]. In general, depression reduces functional, emotional, cognitive and physical capacities needed to recover from coexisting somatic diseases.

Concerning depression in otorhinolaryngologic (ORL) diseases, prior studies mostly report high prevalences. Values range from 10% to 26% [7-11] indicating that patients with ORL are at high risk for depression. This is not surprising considering that characteristics of ORL diseases may entail severe consequences on subjective functioning and everyday quality of life [12].

However it is important to note, that in most published studies only self-report instruments were used to assess depression, e.g., the Hospital Anxiety and Depression Scale [HADS; 7,8]. Those studies that applied a higher diagnostic standard, e.g. the use of diagnostic interviews, mostly referring to head and neck cancer, reported highly divergent prevalence rates depending for example on the subsample of ORL patients [13-15]. In some studies, no standardized instrument was applied at all [9,10], or no information about how depressive diagnoses were determined were reported [11]. This is critical since validity and reliability of epidemiological studies depend largely on the quality of the instrument applied to collect diagnostic information.

The international accepted "gold standard" for diagnosing mental disorders like depression is a semi-structured diagnostic interview conducted by trained personnel. Self-report instruments are often used instead for economical reasons. However, to allow for reliable and sound conclusions in studies on the prevalence of depression, applying the diagnostic gold standard is strongly demanded. Nevertheless, studies using a semi-structured interview to determine the prevalence of depression in ORL are lacking.

Therefore, the present study had two major aims: (a) to determine the prevalence of depression in consecutive ORL inpatients applying a semi-structured diagnostic interview; (b) to compare the pathopsychological and demographic characteristics of depressed and non-depressed ORL inpatients in order to gain further insight into the characteristics of patients suffering from depressive disorders in this population.

## Methods

### Design

This was a cross-sectional study with consecutive inpatients of the department for otorhinolaryngology of a German university hospital. Prevalence of depressive disorders was determined. Depressive status served than as independent variable while measures of mental symptom burden (Somatisation, Obsessive-Compulsive, Interpersonal Sensitivity, Depression, Anxiety, Hostility, Phobic Anxiety, Paranoid Ideation, Psychoticism) were assessed as dependent variables.

### Sample

One-hundred and two consecutive inpatients of the department for otorhinolaryngology of a German university hospital participated voluntarily. Two participants suffering from chronic diseases for more than 30 years were excluded from analyses because this time period exceeded the mean duration of disease in the sample by much more than two standard deviations. Overall mean age of the remaining 100 patients was 38.8 years (SD = 13.9) and 38.0% were female. See table 1 for sample details. Participants took part voluntarily without payment and signed an informed consent prior to testing. General inclusion criteria were German language skills and the ability to concentrate for at least 1 hour. Test administration was conducted by trained personnel. The study was approved by the local ethics committee of the Medical Faculty of the RWTH Aachen University (EK 172/05) and performed according to the Declaration of Helsinki [16].

**Table 1:** Detailed sample description according to ICD-10; multiple diagnoses possible

	N	age (SD)	% female	duration of disease^a ^(SD)	duration of stay^a ^(SD)	Impairment ADL^b ^(SD)	Impairment QoL^c ^(SD)	BDI sum
whole sample	100	38.8 (13.9)	38.0	151.8 (401.4)	8.3 (4.4)	1.9 (1.5)	1.8 (1.3)	6.7 (7.7)
diseases of external ear (H60-H62)	2	47.5 (3.5)	33.3	3.5 (0.7)	5.5 (0.7)	1.5 (2.1)	1.0 (1.4)	4.0 (1.4)
diseases of middle ear & mastoid (H65-H75)	1	30.0 (--)	75.0	4.0 (--)	4.0 (--)	0.0 (--)	0.0 (--)	5.0 (--)
diseases of inner ear (H80-H83)	2	42.0 (7.1)	57.1	12.0 (1.4)	10.5 (7.1)	2.5 (2.1)	3.0 (1.4)	7.0 (7.1)
hearing loss (H90-H91)	11	44.7 (13.2)	63.6	87.6 (241.0)	10.3 (5.3)	2.8 (1.3)	2.3 (1.3)	8.6 (7.9)
diseases of nose and paranasal sinuses (J01, J32, J34)	16	44.1 (17.8)	18.8	222.2 (626.4)	8.6 (5.1)	2.5 (1.4)	1.7 (1.3)	5.2 (4.3)
diseases of mouth, throat & pharynx (J03, J35-J38, K07, K11-K13)	37	34.0 (13.5)	40.5	131.3 (305.2)	8.1 (3.7)	1.3 (1.5)	1.7 (1.5)	7.9 (9.2)

^a ^in days; ^b ^ADL: Activities of Daily Living; ^c ^QoL: Quality of Life

### Procedures

Test sessions took place at admission and started with the conduction of a clinical interview in which the International Diagnostic Checklist for depression [IDCL; 17] was conducted. Afterwards, participants completed a demographic data sheet, and filled in further questionnaires.

### Measures

#### Clinical Interview

Depression was assessed in all participants using a clinical interview in which the International Diagnostic Checklist for depression [IDCL; 17] was employed to verify the diagnosis. The IDCL is a checklist that can be used to make a careful evaluation of the symptoms and classification criteria, and thus help to arrive at precise diagnoses according to the 10^th ^edition of the International Classification of Diseases (ICD-10) criteria for a depressive episode [18]. Persons who conducted the clinical interview were either psychologists or medical students in their last year with a major in psychiatry. All interviewers received one week training in completing this interview consisting of three steps: First, the interview guidelines were presented and the trainee observed a couple of interviews conducted by the principal author. Second, the trainee did role-play interviews with the principal author as "participant". Third, the trainee conducted interviews with real participants supervised by the principal author. If the trainee's diagnoses were in accordance with the trainer's, then the trainee was eligible as an interviewer in the present study.

#### Brief Symptom Inventory (BSI)

The Brief Symptom Inventory is a short form of the Symptom Checklist 90-R [SCL-90-R; 19] and contains 53 items that are Likert-scaled, referring to the previous week, with a range from 0 ("not at all") to 4 ("very much"). The instrument provides information on overall psychological distress. Furthermore, the 53 items of the inventory constitute three composite scores and nine symptom scales (Somatisation, Obsessive-Compulsive, Interpersonal Sensitivity, Depression, Anxiety, Hostility, Phobic Anxiety, Paranoid Ideation, Psychoticism) allowing the calculation of psychopathological profiles. The three composite scores reflect the complete answer pattern of the respondent: the "global severity index" (GSI) measures the overall mental symptom burden, the "positive symptom distress index" (PSDI) measures symptom intensity, and the "positive symptom total" (PST) reflects the total number of the respondent's symptoms. The raw scale and composite scores are transformed to standardized T-scores with a mean of 50 and a standard deviation (SD) of 10. T-scores > 60 reflect heightened mental burden [20].

#### Beck Depression Inventory (BDI)

The BDI [21] contains 21 items. Each item consists of four self-referring statements (e.g. "I am sad"). Item scores range from 0 to 3 and participants are supposed to choose one or more statements per item that represents best their mental state during the last week. A total score > 10 indicates mild to moderate depression and a total score > 18 moderate to severe depression. The BDI has not been validated in ORL inpatients so far, so that all conclusions based on the BDI in this study should be handled with care.

#### Further materials

All participants completed a demographic data sheet. Furthermore, the level of impairment in activities of daily living (ADL) and in quality of life (QoL) was inquired by a 5-point Likert-scale (0 = no impairment, 1 = little, 2 = moderate, 3 = strong, 4 = very strong). Clinical data were taken from medical records. Data were acquired within the scope of a comprehensive research project, so that participants filled in further questionnaires that are reported elsewhere [22].

### Data analysis

The number of depressed and non-depressed ORL patients according to IDCL was determined. Mean age and standard deviations and number of male and female participants in both groups were calculated.

A mulitivariate analysis of variance (MANOVA) with "group" (depressed vs. non-depressed ORL patients) as between-subjects-factor was conducted. Prior to MANOVA, homogeneity of error variances was tested with the Mauchly test for sphericity. Homogeneity of error variances is important in order to interpret the results of MANOVA validly. Therefore, in case of significant results of the Mauchly test and thus violation of homogeneity of error variances, a Greenhouse-Geisser-correction was conducted. Effect sizes according to Hedges and Olkin [23] were calculated. Effect sizes amend significance tests reasonably since they allow for an estimation of the clinical relevance of empirical differences which is less sensitive to sample size than significance tests (e.g., t-tests). Cohen [24] recommended to interpret an effect size *d *of .20< *d *≤.50 as small, an effect size of .50< *d *≤.80 as medium and an effect size of *d *≥.80 as large. Following the recommendations of Dunlap [25] in the present study effect sizes were calculated for independent variables instead of dependent variables because effect sizes for dependent variables often overestimate the actual size of effect.

Bivariate correlation analyses were performed to further characterize those patients who were assigned a depressive disorder. Classificatory (IDCL) as well as dimensional (BDI) information about the depressive status of the participants were correlated with age, gender, marital status, levels of impairment in ADL and in QoL, duration of disease, and duration of stay in hospital. All analyses were performed using SPSS 17 for Windows.

## Results

### Prevalence of depression

Considering the remaining sample (N = 100), the semi-structured interview based on the IDCL-checklist for depression revealed that 21 participants suffered from a depressive disorder, which corresponds to a prevalence rate of 21.0%. The mean score of BDI was 14.2 (SD = 12.2) in the depressed and 4.8 (SD = 4.2) in the non-depressed group. In fourteen patients (14%) a single depressive episode was found, 5 patients (5%) exhibited a recurrent depressive disorder. Nearly forty-eight percent (47.6%) of those patients with any depressive disorder were women (table 2). The mean age of patients with depression was 39.4 years (SD = 11.7) and the mean age of those without depression was 38.6 years (SD = 14.5; see table 2 for details).

**Table 2:** Prevalence rates according to ICD-10, divided by gender and diagnosis

	all patients		female		male	
**Diagnosis**	**frequency**	**percentage**	**frequency**	**percentage**	**frequency**	**percentage**

no depressive disorder	79	79.0	28	35.4	51	64.6
any depressive disorder	21	21.0	10	47.6	11	52.4
F31.3: Bipolar affective disorder, current episode mild or moderate depression	1	1.0	1	100.0	0	0.0
F31.4: Bipolar affective disorder, current episode severe depression	1	1.0	1	100.0	0	0.0
F32.0: Mild depressive episode	8	8.0	2	25.0	6	75.0
F32.1: Moderate depressive episode	4	4.0	1	25.0	3	75.0
F32.2: Severe depressive episode without psychotic symptoms	1	1.0	0	0.0	1	100.0
F32.4: depressive episode, in partial remission	1	1.0	0	0.0	1	100.0
F33.1: Recurrent depressive disorder, current episode moderate	2	2.0	2	100.0	0	0.0
F33.2: Recurrent depressive disorder, current episode severe without psychotic symptoms	3	3.0	3	100.0	0	0.0

total	100	100	38	38.0	62	62.0

### Psychopathological and demographic characteristics of depressed vs. non-depressed ORL patients

The Mauchly test for sphericity revealed inhomogeneity of error variances (Mauchly-W = .013; p < .001). Thus, a Greenhouse-Geisser correction was performed prior to MANOVA. Depressed patients had higher scores than non-depressed patients on all scales (Somatisation, Obsessive-Compulsive, Interpersonal Sensitivity, Depression, Anxiety, Hostility, Phobic Anxiety, Paranoid Ideation, Psychoticism) and all three composite scores GSI, PSDI and PST. However, the corresponding main effect "group" was not significant (F = .85; df = 1; p = .60).

Univariate tests of between subjects effects showed significant differences between depressed and non-depressed patients on the scales "depression" (F = 4.53; df = 1; p = .04) and "anxiety" (F = 4.84; df = 1; p = .03) and for the global score PSDI (F = 4.38; df = 1; p = .04). Analyses of effect sizes showed relevant effect sizes between depressed and non-depressed patients of medium size for "interpersonal sensitivity", "depression", "anxiety", "phobic anxiety", "psychoticism" and the PSDI (see table 3). Most mean scores in both groups were within the range of normal mental symptom burden (T-value = 50 +/- 10). Only the mean score on the composite score PST in the depressed group was greater than a T-value of 60.

**Table 3:** effect sizes between depressed and non-depressed patients for all BSI scores

BSI score	nondepressed			depressed			d	SE
	**mean**	**n**	**SD**	**mean**	**n**	**SD**		

somatisation	54.1	72	11.6	58.1	19	11.7	-0.34	0.26
obsessive-compulsive	47.6	73	10.9	53.2	19	15.6	-0.46	0.26
interpersonal sensitivity	47.3	74	9.6	54.6	19	15.2	-0.66	0.26
depression	48.2	74	8.6	54.9	19	13.8	-0.68	0.26
anxiety	50.1	74	10.9	58.1	18	13.9	-0.69	0.27
hostility	49.3	74	10.2	52.3	18	11.9	-0.28	0.26
phobic anxiety	49.9	74	9.3	55.5	19	9.6	-0.59	0.26
paranoid ideation	50.3	73	10.4	54.8	19	13.1	-0.40	0.26
psychoticism	49.4	72	9.7	56.5	19	13.1	-0.67	0.26
GSI	49.4	68	12.5	54.8	17	17.8	-0.39	0.27
PSDI	54.8	65	12.2	61.7	16	10.2	-0.58	0.28
PST	48.0	68	13.1	53.8	17	18.6	-0.40	0.27

Note: d = effect size, bias corrected according to Hedges & Olkin (1985); SE = standard error of effect size estimate.

Bivariate correlation analyses between classificatory (IDCL) as well as dimensional (BDI) information about the depressive status of the participants, age, gender, marital status, levels of impairment in ADL and QoL, duration of disease, and duration of stay in hospital were performed. Gender correlated significantly with dimensional information about the depression status (BDI) of participants (r = .25; p = .013) which persisted when controlling for age: depressed patients were slightly more likely to be male. No further significant correlations were found.

## Discussion

The calculated overall prevalence of depression was 21.0%. Gender was significantly correlated with classificatory information about the patients' affective status. This is largely in accordance with published data on the prevalence of depression in this patient collective [7-10]. The fact that slightly more male than female patients exhibited depressive symptoms might be explainable by the higher base rate of male patients (62%) in the study sample. Generally, the present study confirms results of previous investigations. However, a more reliable technique for determination of the diagnostic information was applied here (semi-structured interview) as compared to most other published studies on the prevalence of depression in ORL, so that we further substantiated previous findings. Above, analyses were conducted with data of an unselected sample of consecutive ORL inpatients. Therefore, the prevalence rate reported here reflects more directly the situation that clinicians encounter in their routine clinical practice than most previous studies did.

Depressed patients reported to suffer more from symptoms referring to anxiety, depression, interpersonal sensitivity, psychoticism, and phobic anxiety than non-depressed patients. Differences were moderate in terms of effect sizes. However, total symptom burden on most BSI-scales was only moderate in *both *groups. Nevertheless, all mean scores coincided with a huge standard deviation indicating that patients *within *both groups differed largely in terms of symptom burden.

The present study largely replicated the picture of depression that is known from a multiplicity of studies on this disease in other samples: It is a well known fact that depression often co-occurs with anxiety and obsessive-compulsive or phobic disorders, respectively [26-28].

The prevalence rate of depression found in the present study is largely consistent with prevalence rates of depression reported in other medical illnesses, e.g., cardiac diseases (17-27%) [29], cerebrovascular diseases (14-19%) [30], obesity (20-30%) [31], cancer (22-29%) [32] or HIV/AIDS (5-20%) [33]. Simultaneously, it is considerably higher than the prevalence found in the general population (10.3%) [2]. Evans [5] assembled studies that indicate a bi-directional relation between depression and comorbid medical illnesses: severe medical illness is an accepted risk factor for developing a depressive disorder. However, at the same time, it is under debate whether depression might be a causal factor itself in the development and course of medical illnesses, e.g., in cardiac diseases [34]. In this light, the relatively high prevalence rate of depression found in the present study might be interpreted as further evidence for a strong link between depression and medical illnesses of ORL in particular. Thus, one could assume that depression might impair treatment and outcome of otolaryngologic diseases like the data of Bhattacharyya & Wasan suggest [7]. Still, further research is needed, and especially longitudinally designed studies are required to gain further insight into the complex relation of depression and physical disease.

In general, the conduction of a full semi-structured diagnostic interview to assess mood in ORL-patients would be desirable. However, since this technique is laborious and time-consuming for both, the patient and the investigator, it is not likely that it will become routine diagnostic practice in ORL clinics. Still, given the general impact of depression in patients with various physical diseases [34,35] a routine screening for depression with more economical instruments is highly demanded. Therefore, future studies should engage in expanding our body of knowledge about the screening performance (sensitivity, specificity) of existing self-report instruments for depression (e.g., BDI) in this patient group. A new instrument that shows promising psychometric quality in both patients with physical and mental illnesses is the Rasch-based Depression Screening (DESC), which was developed on the basis of data from patients with mental, cardiologic, and otorhinolaryngologic diseases [36].

A limitation of the present study is that no detailed data on those patients who declined participation is available. Patients were approached at admission if their treating physicians considered them eligible and most (approximately > 80%) agreed to participate. Nevertheless, a potential bias of over- or underreporting of depression can not be ruled out. Overreporting would occur if those persons with more severe symptoms of depression would have been more likely to participate - e.g., because they felt that study aims were important for people in their present situation. Underreporting would have occurred if those persons with more severe depression would have been more likely to decline participation - e.g., because symptom burden was too high. Both directions of bias may be present in most studies of this kind and have to be kept in mind when interpreting the current results.

Another limitation is that although published research suggests that especially diagnoses like head and neck cancer might be related to elevated depression [14,32], limited sample size made it impossible to report reliable prevalence rates for ORL subsamples with different diagnoses so that we decided to report prevalence data only divided by gender.

## Conclusion

The results of our study call for carrying on in engaging in research about depression in ORL inpatients and further intensifying collaborative health care in a multidisciplinary setting to foster optimal outcome and treatment of both, the physical and psychic disorder.

## List of abbreviations used

ADL: activities of daily living; BDI: Beck Depression Inventory; BSI: Brief Symptom Inventory; DESC: Rasch-based Depression Screening; GSI: global severity index; HADS: Hospital Anxiety and Depression Scale; ICD-10: International Classification of Diseases 10^th ^Revision; IDCL: International Diagnostic Checklist for depression; MANOVA: analysis of variance for repeated measures; ORL: otorhinolaryngology; PSDI: positive symptom distress index; PST: positive symptom total; QoL: quality of life; SCL-90-R: Symptom Checklist 90-R; SD: standard deviation

## Competing interests

The authors declare that they have no competing interests.

## Authors' contributions

TF contributed to conception and design of the study, conducted the statistical analysis and wrote the manuscript. CN participated in the analysis and interpretation of the data. MW participated in the design of the study and the statistical analysis. MW and TV participated in the design of the study and coordinated the data acquisition. SG has been involved in drafting and revising the manuscript, and coordinated the study and data acquisition. MB contributed to the analysis and interpretation of the data. All authors read and approved the final manuscript.

## Pre-publication history

The pre-publication history for this paper can be accessed here:

http://www.biomedcentral.com/1472-6815/11/7/prepub
